# Transcutaneous auricular vagus nerve stimulation in anesthetized mice induces antidepressant effects by activating dopaminergic neurons in the ventral tegmental area

**DOI:** 10.1186/s13041-024-01162-x

**Published:** 2024-11-27

**Authors:** Tae-Yong Choi, Jeongseop Kim, Ja Wook Koo

**Affiliations:** 1 Emotion, Cognition and Behavior Research Group, Korea Brain Research Institute, Daegu, 41062 Republic of Korea; 2 Department of Brain Sciences, Daegu Gyeongbuk Institute of Science and Technology, Daegu, 42988 Republic of Korea

**Keywords:** Depression, Transcutaneous auricular vagus nerve stimulation, Antidepressant, Ventral tegmental area, Dopamine, Chronic social defeat stress, Forced swim test, Fiber photometry

## Abstract

Depression, a prevalent neuropsychiatric disorder, involves the dysregulation of neurotransmitters such as dopamine (DA). The restoration of DA balance is a pivotal therapeutic target for this condition. Recent studies have indicated that both antidepressant medications and non-pharmacological treatments, such as transcutaneous auricular vagus nerve stimulation (taVNS), can promote recovery from depressive symptoms. Despite the promise of taVNS as a non-invasive depression therapy, its precise mechanism remains unclear. We hypothesized that taVNS exerts antidepressant effects by modulating the DAergic system. To investigate this, we conducted experiments demonstrating that taVNS in anesthetized mice reduced depressive-like behaviors. However, this effect was abolished when DA neurons in the ventral tegmental area (VTA^DA^) were inhibited. Additionally, taVNS in anesthetized mice enhanced VTA^DA^ activity, providing further evidence to support its antidepressant effects. Overall, our findings suggest that taVNS alleviates depression by augmenting VTA^DA^ activity, thereby contributing to a more comprehensive understanding of its therapeutic mechanisms.

**Supplementary Information:**

The online version contains supplementary material available at 10.1186/s13041-024-01162-x.

## Introduction

Depression, or major depressive disorder (MDD), is a prevalent and severe neuropsychiatric disease characterized by persistent low mood and diminished interest or pleasure in activities. Although numerous factors can contribute to depression, its principal determinants remain elusive. Emerging evidence has indicated that the dysregulation of neurotransmitters, notably dopamine (DA), particularly within brain regions governing emotions, cognition, sleep, and appetite, plays a pivotal role in its etiology [1]. As such, restoring the balance in brain chemistry may alleviate depression.

It has been widely recognized that the activity of DA neurons in the ventral tegmental area (VTA^DA^) and DA release to various brain regions regulate mood-related behaviors. Indeed, research has shown that stress or aversive stimuli suppress VTA^DA^ activity [2], while the optogenetic inhibition of VTA^DA^ leads to a depressive-like phenotype. Conversely, phasic photoactivation of VTA^DA^ has been shown to mitigate chronic stress-induced depressive symptoms [3]. Overall, these findings underscore the importance of regulating VTA^DA^ as a primary therapeutic approach for depression.

Numerous antidepressant medications, including dopamine agonists, dopamine reuptake inhibitors, and norepinephrine and dopamine reuptake inhibitors, as well as non-pharmacological therapies, such as electroconvulsive therapy, repetitive transcranial magnetic stimulation, and vagus nerve stimulation (VNS), have all been employed in the treatment of depression, to regulate the DAergic system [4]. In particular, non-invasive VNS modalities, such as transcutaneous auricular VNS (taVNS), are increasingly being used to treat depression owing to their multifaceted advantages [5]. However, the precise mechanism by which taVNS mitigates depression remains unclear.

## Results

**Fig. 1:**
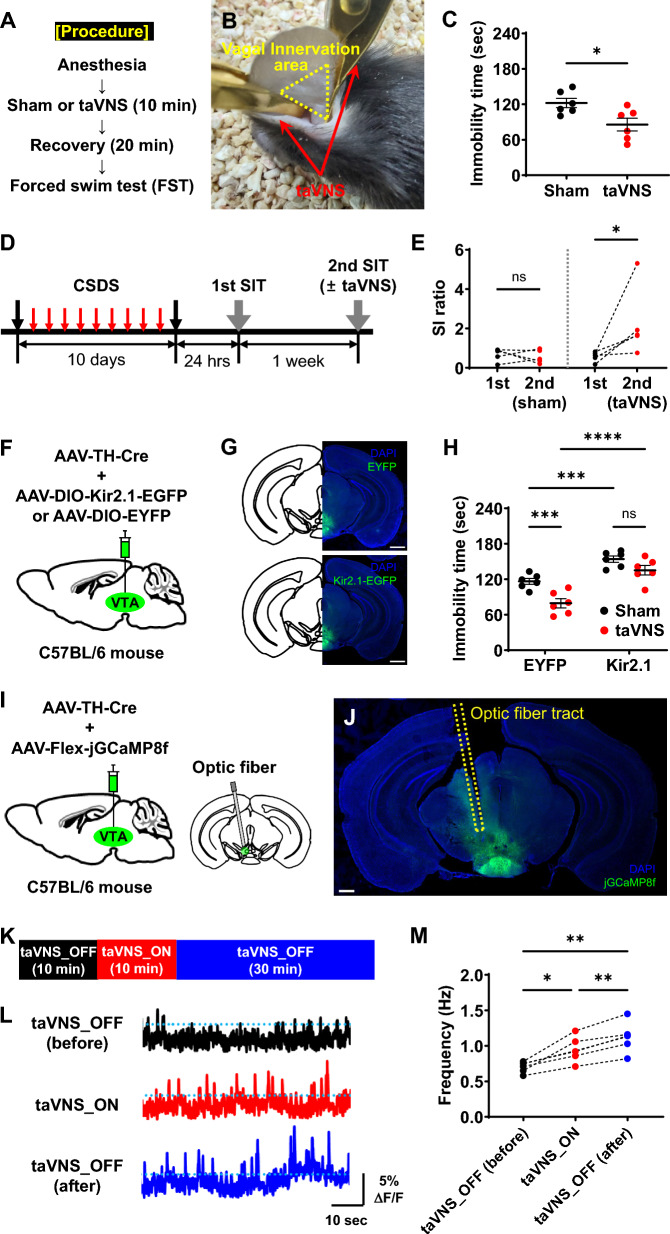
taVNS induces antidepressant effects by increasing VTA^DA^ activity. **A** The FST procedure without (i.e., off-site stimulation or sham control) or with taVNS intervention. **B** A representative image of a mouse treated with taVNS, followed by the cymba and cavum concha of the vagus innervation area in the ear. **C** Immobility time during the FST. Animals were treated with or without taVNS. Values are shown as the mean ± standard error of the mean (SEM) (n = 6 mice per group; Student’s t-test [unpaired, two-tailed]; *p < 0.05). **D** Schematic diagram depicting the experimental procedure for CSDS and social interaction test (SIT) with or without taVNS. **E** taVNS significantly reversed CSDS-induced social avoidance. (n = 5 mice per group; Student’s t-test [paired, two-tailed]; ns, not significant; *p < 0.05). **F** Schematic illustration of viral injection to inhibit VTA^DA^. **G** Representative images of brain sections injected with AAV-rTH-Cre and AAV-DIO-EYFP (top) or AAV-DIO-Kir2.1-EGFP (bottom) into the VTA. DAPI, blue; EYFP (top) or Kir2.1-EGFP (bottom), green. Scale bars, 1000 μm. **H** Immobility time during the FST. Control and VTA^DA^-inhibited mice were treated with or without taVNS. Values are shown as mean ± SEM (n = 6 mice per group; Two-way ANOVA with Fisher’s LSD multiple comparisons test; ns, not significant; ***p < 0.001; ****p < 0.0001). **I** Schematic illustration of viral injection and optic fiber implantation to measure VTA^DA^ activity using fiber photometry. **J** A representative image of a brain section infected with AAV-rTH-Cre and AAV-Flex-jGCaMP8f in the VTA. A fiber optic cannula was implanted above the viral injection site. DAPI, blue; jGCaMP8f, green. Scale bar, 500 μm. **K** Procedure for fiber photometry recordings with taVNS. **L** Representative Ca^2+^ traces from a single animal. Data are presented as the percentage change in fluorescence over the mean fluorescence (ΔF/F). Top, taVNS_OFF (before, 10 min); middle, taVNS_ON (10 min); bottom, taVNS_OFF (after, 30 min). **M** Peak analysis of Ca.^2+^ imaging traces. (n = 6 mice; repeated-measures one-way ANOVA with Tukey’s multiple comparisons test; *p < 0.05; **p < 0.01)

We hypothesized that taVNS exerts its antidepressant effects by activating VTA^DA^ activity. Initially, we investigated whether taVNS induced this effect through conduction of the forced swim test (FST), a standard rodent behavioral test commonly used to assess the efficacy of antidepressant drugs or treatments in eliciting or preventing depressive-like states, 20 min after sham (i.e., off-site stimulation) or taVNS intervention in anesthetized mice (Fig. 1A, B) [6]. As a result, taVNS, but not sham treatment, in anesthetized mice led to a reduction in immobility time during the FST, indicating its antidepressant effect (Fig. 1C).

Additionally, we explored whether taVNS could alleviate depressive symptoms induced by chronic social defeat stress (CSDS), an ethologically validated animal model of depression [7, 8]. CSDS markedly diminished social interaction with an aggressor mouse only in susceptible mice, while resilient mice were unaffected (Fig. S1). Notably, our findings revealed that taVNS treatment in an anesthetized state effectively reversed CSDS-induced social avoidance (Fig. 1D, E). These results suggest that taVNS may exert anti-stress or antidepressant effects under both normal and depressive conditions.

Next, we examined whether the antidepressant effects of taVNS in anesthetized mice are mediated by VTA^DA^ activity. To investigate this, we performed the FST with or without taVNS in mice that received AAV-rTH-Cre [9] and AAV-DIO-Kir2.1-EGFP [10] into the VTA to inhibit VTA^DA^ (Fig. 1F, G). As anticipated, inhibition of VTA^DA^ resulted in increased immobility time during the FST (Fig. 1H), consistent with findings from a previous study [3]. Importantly, taVNS in an anesthetized state did not reduce immobility time in these mice (Fig. 1H). These results indicate that the antidepressant effects of taVNS are mediated through VTA^DA^ activity.

Finally, we investigated whether taVNS influenced VTA^DA^ activity. To accomplish this, we measured VTA^DA^ activity via fiber photometry in mice that received AAV-rTH-Cre and AAV-Flex-jGCaMP8f [11] and implanted optic fibers into the VTA (Fig. 1I, J). Overall, we found that taVNS in anesthetized mice increased the frequency of Ca^2+^ transients of VTA^DA^, suggesting an increase in the average activity of VTA^DA^ (Fig. 1K–M). Interestingly, the increased activity of VTA^DA^ induced by taVNS was further increased for 30 min following taVNS (Fig. 1K–M). These results indicate that the potentiation of VTA^DA^ activity by taVNS resulted in an antidepressant effect.

## Discussion

The vagus nerve, also known as the tenth cranial nerve, is one of the 12 cranial nerves that emerge directly from the brain. It plays a crucial role in the parasympathetic nervous system, which regulates numerous unconscious bodily functions such as heart rate, digestion, and respiratory rate. Further, the vagus nerve has been implicated in the body’s response to stress. The activation of the vagus nerve can induce relaxation and calmness [12]. Consequently, despite the use of VNS for depression treatment, its precise mechanisms remain incompletely understood.

Information from several peripheral organs is relayed to the brain, particularly to the nucleus of the solitary tract (NTS) in the brainstem. The NTS is interconnected with various brain regions responsible for synthesizing specific neurotransmitters, including the locus coeruleus, which produces norepinephrine, the dorsal raphe nucleus, which synthesizes serotonin, and the VTA, which produces DA [13]. This suggests that VNS can be used to treat various neuropsychiatric disorders, including depression, by modulating these neurotransmitter systems throughout the brain.

Although previous studies have reported that invasively implanted VNS induces an antidepressant effect [14, 15] and activates VTA^DA^ [16], whether and how non-invasive taVNS elicits an antidepressant effect currently remains unclear. Invasive VNS is effective for treating various neuropsychiatric disorders and has sustained therapeutic effects. However, it carries the risks of surgery, including infection, pain, and high cost [17]. Conversely, taVNS offers a non-invasive approach without the need for surgical procedures, along with high levels of safety, ease of use, and relatively low cost [18].

Overall, our findings suggest that taVNS in anesthetized mice triggers antidepressant effects by enhancing VTA^DA^ activity. Further investigation is required to identify the specific brain regions where the heightened VTA^DA^ activity induced by taVNS results in increased DA release and whether this contributes to the antidepressant effect. Furthermore, preclinical validation in animal models of depression is essential to confirm whether taVNS is effective for clinical application based on the mechanisms we have elucidated.

## Supplementary Information

Additional file 1.

## Data Availability

The datasets used and/or analyzed during the current study are available from the corresponding authors on reasonable request.

## Abbreviations

AAV: Adeno-associated virus
CSDS: Chronic social defeat stress
DA: Dopamine
FST: Forced swim test
MDD: Major depressive disorder
SI: Social interaction
SIT: Social interaction test
taVNS: Transcutaneous auricular vagus nerve stimulation
TH: Tyrosine hydroxylase
VNS: Vagus nerve stimulation
VTA: Ventral tegmental area
