# Supplementary material for: Transcutaneous auricular vagus nerve stimulation in anesthetized mice induces antidepressant effects by activating dopaminergic neurons in the ventral tegmental area
Source: Mol Brain. 2024 Nov 27;17:86. doi: 10.1186/s13041-024-01162-x (PMC11600629; doi:10.1186/s13041-024-01162-x)
Supplement: Supplementary file 1 — Additional file 1. [file 13041_2024_1162_MOESM1_ESM.docx]

**Additional file**

Transcutaneous auricular vagus nerve stimulation in anesthetized mice induces antidepressant effects by activating dopaminergic neurons in the ventral tegmental area.

Tae-Yong Choi^1#*^, Jeongseop Kim^1#^, Ja Wook Koo^1,2*^

^1^Emotion, Cognition and Behavior Research Group, Korea Brain Research Institute, Daegu 41062, Republic of Korea

^2^Department of Brain Sciences, Daegu Gyeongbuk Institute of Science and Technology, Daegu 42988, Republic of Korea

^#^Equal contribution

^*^Correspondence to:

Tae-Yong Choi, Ph.D. (tychoi5667@kbri.re.kr); Ja Wook Koo, Ph.D. (jawook.koo@kbri.re.kr)

**This PDF file includes:**

Supplementary Materials and Methods

Supplementary Figures and Tables: Figure S1, Table S1

**Supplementary Materials and Methods**

**Mice**

Adult (over 2 months old) male C57BL/6N mice (25-30 g, Orient Bio, South Korea) were used for this study. Animals were maintained in the Laboratory Animal Center at KBRI with a specific pathogen-free barrier under a 12-h light/dark cycle (lights on at 08:00 am), the temperature at 22 ± 2 °C and the humidity at 50 ± 10%, and given access to food and water ad libitum. All mice were exposed to vivarium conditions for at least 1 week before behavioral tests. All surgical manipulations and behavioral tests were carried out in the animal facility during their light on period. Every effort was made to minimize the number of animals used in this study and reduce animal suffering. Animals with any health abnormalities, such as body weight loss, fur loss and wounds, were excluded from the experiment.

**Stereotaxic surgeries**

Stereotaxic surgeries were conducted as described previously [1]. In brief, mice were deeply anesthetized by an intraperitoneal injection of 0.1M phosphate-buffered saline (PBS) containing ketamine (100 mg/kg) and xylazine (10 mg/kg). After confirming deep anesthesia, mice were fixed on a stereotaxic apparatus (Stoelting Co.). Fine surgical scissors were used to make a midline incision to expose the skull, and a microdrill was used to make the craniotomies above the locations where viral vectors were injected or fiber optic cannula was implanted. For all injections, a 5-μL microsyringe (7641-01, Hamilton) connected with a 33-gauge needle (7762-06, Hamilton) was used. Viral vectors were injected at a rate of 100 nL/min, and the needle was kept in place for an additional 10 min to allow for viral dispersion before being removed. The scalp incision was closed with a surgical suture and tissue adhesive (Vetbont, 3M). For implanting fiber optic cannula, dental cement was applied to secure them. All stereotaxic coordinates were measured relative to bregma. After surgery, mice recovered in a clean cage under a heat lamp until awakening from anesthesia. Before undergoing subsequent operations or behavioral tests, mice were given a minimum of two weeks to recuperate.

For inhibiting VTA^DA^ (Fig. 1D-F), C57BL/6N mice were bilaterally injected 800 nL of a mixture of two viruses (AAV9-rTH-Cre (107788-AAV9, Addgene) and AAVdj-EF1a-DIO-Kir2.1-EGFP (PT-1401, BrainVTA) or AAV5-EF1a-DIO-EYFP (UNC Vector Core), ratio, 1:1) into the VTA (anteroposterior (AP), -3.2 mm; mediolateral (ML), ±1.2 mm; dorsoventral (DV), -4.6 mm; angle, 10º).

For measuring VTA^DA^ activity using fiber photometry experiments (Fig. 1I-K), 800 mL of a mixture of two viruses (AAV9-rTH-Cre and AAV9-Syn-Flex-jGCaMP8f (162379-AAV9, Addgene), ratio, 1:1) were injected into the left VTA. Fiber optic cannula (400 μm core diameter, 0.50 NA; RWD Life Science) was implanted 200 μm above the viral injection coordinates of the VTA on the same day or several days after the virus injection.

**Transcutaneous auricular vagus nerve stimulation (taVNS)**

The taVNS was conducted according to previously published protocol with minor modifications [2]. Two electrodes coated with a gold-plated platinum hook were placed on the auricular concha of the left ear. Electrodes were connected to SIU-91 stimulus isolator (Cygnus). They are connected to Multiclamp700B (Molecular Devices) that generates bipolar electrical pulses by pClamp10 (Molecular Devices). Both the cymba and cavum conchae of the auricular were biphasically stimulated with the same parameter (output current, 0.2 mA; pulse width, 0.2 ms; pulse frequency, 25 Hz; time, 10 min).

Stimulation was performed under isoflurane anesthesia (2% induction and 1.5% maintenance) in 0.8 L / min O_2_. The off-site stimulation (sham) was delivered to the tip of the ear helix, which was innervated by the cervical spinal nerve but not by the auricular branch of the vagus nerve (ABVN).

**Forced swim test (FST)**

The FST was conducted according to previously published protocol [3]. Before the test, all mice were placed in a 2000-mL Pyrex glass beaker containing 1200 mL of water for 6 min, and the water was changed between subjects. A video camera mounted on the side of the beakers was used to record all the test sessions. An observer who was unaware of the treatment evaluated and rated the videotapes. Immobility was assessed during the last 4 min of the test.

**Chronic social defeat stress (CSDS)**

The CSDS was conducted following an established protocol, as previously described [4]. Each C57BL/6N mouse was subjected to 10 min of physical aggression by a male CD-1 (or aggressor) mouse. Following this session, the defeated C57BL/6N mouse was housed overnight in the same cage as the CD-1 mouse, separated by a transparent, perforated divider that allowed sensory but not physical contact. This procedure was repeated for 10 consecutive days, with a new aggressor mouse introduced each day. Control C57BL/6N mice were housed in similar two-compartment cages, with different littermates daily. The CSDS protocol was carried out during the light cycle between 14:00 and 17:00.

**Social interaction test (SIT)**

One day after the final CSDS session, a SIT was administered to the experimental C57BL/6N mice. The test consisted of two sessions, in which the defeated or control mice explored a square arena for 2.5 min each, either in the absence or presence of a novel CD-1 mouse. During the first session, an empty wire mesh enclosure was positioned against one of the arena walls to assess baseline exploration. In the second session, an unfamiliar CD-1 mouse was placed inside the wire mesh enclosure, designated as the social interaction zone. The time spent interacting with the social target was recorded in both sessions. If necessary, the social interaction ratio (interaction time with the social target present/time in the interaction zone without the social target) was calculated to classify mice as either susceptible (social interaction ratio < 1) and resilient (social interaction ratio ≥ 1). The same procedure was repeated one week after the first SIT, following a 10 min of taVNS or sham and a subsequent 20 min of recovery, to eliminate the effect of first SIT. The time spent in the interaction zone was measured using a digital video system and SMART 3.0 (Panlab, Barcelona, Spain) video tracking software.

**Fiber photometry**

All fiber photometry experiments were conducted as described previously with minor modifications [1]. Doric Fiber Photometry System (Doric Lenses) was used. The two connectorized LEDs (CLEDs, 405 nm regulated at 208.616 Hz for calcium-independent signals and 465 nm regulated at 572.205 Hz for calcium-dependent signals) were controlled by the fiber photometry console via the LED driver. Each CLED was coupled to Integrated Fluorescence Mini Cube with 4 ports (ilFMC4_IE(400-410)_E(460-490)-F(500-550)_S) via an attenuating patch cord, and S port of the Mini Cube was coupled to a mono fiber-optic patchcord to deliver the excitation light to and to receive emitted light from mice that were deeply anesthetized by an intraperitoneal injection of 0.1M phosphate-buffered saline (PBS) containing ketamine (100 mg/kg) and xylazine (10 mg/kg). The F(500-550) port of the Mini Cube was coupled to the photodetector (Fluorescence Detector Amplifier, Doric Lenses) that was coupled to an analog port of the fiber photometry console. Fiber photometry signals were recorded by Doric Neuroscience Studio (Ver. 5.4.1.23) through the Lock-In mode and a sampling rate of 12.0 kS/s. All recorded signals were decimated by a factor of 100.

Fiber photometry data were analyzed using an open-source analysis package, named photometry modular analysis (pMAT) [5]. Peak analysis to determine frequency of the Ca^2+^ transient signals was done by determining the median average deviation (MAD) of the corrected/normalized data set and peaks were identified as events that exceeded the MAD by 2.91 deviations [6].

**Histology**

Histological experiments were conducted as described previously [1]. After all experiments, we confirmed the location of viral expression or optic fiber tract and excluded the off-target. To verify it, mice were deeply anesthetized with CO_2_ and transcardially perfused with 0.1 M phosphate-buffered saline (PBS) and 4% (wt/vol) paraformaldehyde (PFA) in PBS. Brains were removed, post-fixed overnight in 4% PFA, and equilibrated in 30% sucrose in PBS at 4°C. 50 μm thick coronal sections were cut on a cryocut microtome (CM1860, Leica Biosystems) at -20°C, washed with PBS, and mounted on slide glasses (Muto pure chemicals) and cover-slipped with VECTASIELD HardSet Antifade Mounting Medium with DAPI (H-1500, Vector Laboratories). Images were acquired using a Pannoramic Scan II with a 20X objective lens (3D Histech).

**Statistical analysis**

The number of biological replicates was reported, and several internal replications are present in the study. Animals were randomly assigned to treatment groups. Every experiment used anonymous samples, and the experimenters were not informed of the experimental conditions of the animals. No data were excluded after analyses. Statistical analyses were conducted using GraphPad Prism 10. For n sizes, the number of animals were provided in Figure legends. Data are presented as mean ± the standard error of the mean (SEM) or individual plots. Comprehensive information on statistical analysis is included in the Figure legends and Table S1.

**References for Materials and Methods**

1. Choi TY, Jeon H, Jeong S, Kim EJ, Kim J, Jeong YH, et al. Distinct prefrontal projection activity and transcriptional state conversely orchestrate social competition and hierarchy. Neuron. 2024;112(4):611-627.e8. DOI: 10.1016/j.neuron.2023.11.012.
2. Go YY, Ju WM, Lee CM, Chae SW, Song JJ. Different transcutaneous auricular vagus nerve stimulation parameters modulate the anti-inflammatory effects on lipopolysaccharide-induced acute inflammation in mice. Biomedicines. 2022:10(2):247. DOI: 10.3390/biomedicines10020247.
3. Kim J, Seol S, Kim TE, Lee J, Koo JW, Kang HJ. Synaptotagmin-4 induces anhedonic responses to chronic stress via BDNF signaling in the medial prefrontal cortex. Exp. Mol. Med. 2024;56(2):329-343. DOI: 10.1038/s12276-024-01156-8.
4. Kim J, Kang S, Choi TY, Chang KA, Koo JW. Metabotropic glutamate receptor 5 in amygdala target neurons regulates susceptibility to chronic social stress. Biol. Psychiatry. 2022; 92(2):104-115. DOI: 10.1016/j.biopsych.2022.01.006.
5. Bruno CA, O’Brien C, Bryant S, Mejaes JI, Estrin DJ, et al. pMAT: An open-source software suite for the analysis of fiber photometry data. Pharmacol. Biochem. Behav. 2021;201:173093. DOI: 10.1016/j.pbb.2020.173093.
6. Calipari ES, Bagot RC, Purushothaman I, Davidson TJ, Yorgason JT, Pena CJ, et al. In vivo imaging identifies temporal signature of D1 and D2 medium spiny neurons in cocaine reward. Proc. Natl. Acad. Sci. USA. 2016;113(10):2726-2731. DOI: 10.1073/pnas.1521238113.

**Supplementary Figures and Tables**

**
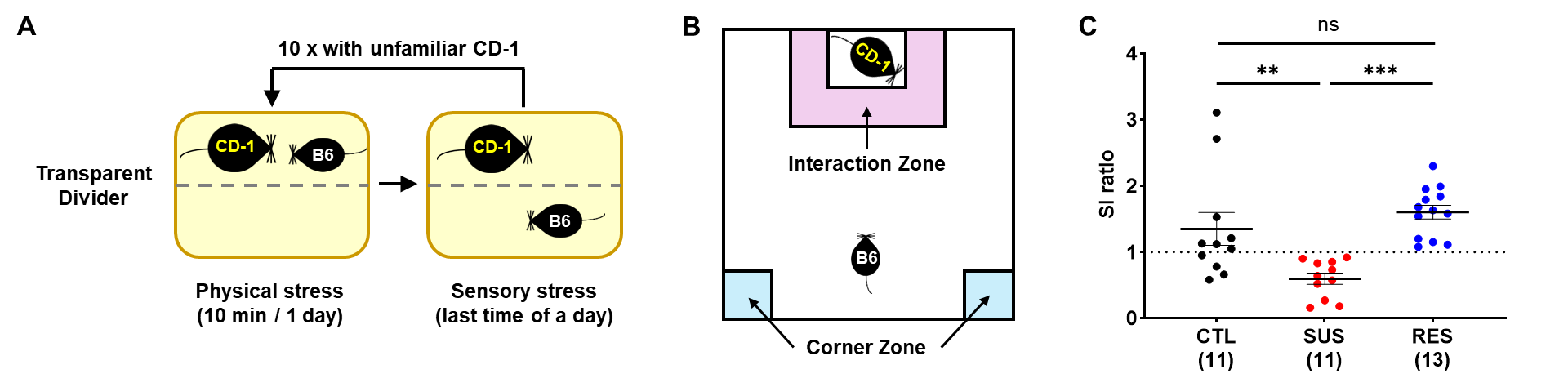
**

**Figure S1.** CSDS produces social avoidance in a small fraction of mice (i.e., susceptible).

1. Experimental schematics of CSDS.
2. Social interaction (SI) testing apparatus depicting the interaction zone (pink) and corner zone (blue).
3. Distribution of SI ratio in control (CTL) and defeated mice. Defeated mice that showed social avoidance (SI ratio < 1) were categorized as susceptible (SUS), and mice that showed social preference (SI ratio ≥ 1) were categorized as resilient (RES). (n = 11 mice (CTL), 11 (SUS) and 13 (RES); One-way ANOVA with Tukey’s multiple comparisons test; ns, not significant; **p < 0.01; ***p < 0.001).

**Table S1.** Summary of statistical analyses.
